# Sodium Alginate Modifications: A Critical Review of Current Strategies and Emerging Applications

**DOI:** 10.3390/foods14223931

**Published:** 2025-11-17

**Authors:** Wenning Wang, Yuanyuan Huang, Yun Pan, Mokhtar Dabbour, Chunhua Dai, Man Zhou, Ronghai He

**Affiliations:** 1 School of Food and Biological Engineering, Jiangsu University, Zhenjiang 212013, China; 2222418067@stmail.ujs.edu.cn (W.W.); 2112318160@stmail.ujs.edu.cn (Y.H.); panyun0213@163.com (Y.P.); manzhou@ujs.edu.cn (M.Z.); heronghai1971@126.com (R.H.); 2 Department of Agricultural and Biosystems Engineering, Faculty of Agriculture, Benha University, Moshtohor, Qaluobia P.O. Box 13736, Egypt; mokhtar.dabbour@fagr.bu.edu.eg; 3 Institute of Food Physical Processing, Jiangsu University, Zhenjiang 212013, China

**Keywords:** sodium alginate, modification method, physical process technology, application

## Abstract

Sodium alginate, a natural anionic polysaccharide, exhibits broad potential applications in food, biomedicine, and environmental engineering due to its favorable biocompatibility, degradability, and functional tunability. This review systematically summarizes its chemical structure, physicochemical characteristics, sources, and extraction methods. It also focused on modification strategies, including chemical approaches (e.g., esterification, oxidation, sulfation, graft copolymerization), physical methods (composite modification, irradiation cross-linking, ultrasound treatment), and biological (e.g., enzyme regulation), and elucidated their underlying mechanisms. In the context of food science, special emphasis is placed on food-compatible chemistries and mild modification routes (such as phenolic crosslinking, enzyme-assisted coupling, and other green reactions) that enable the development of edible films, coatings, and functional carriers, while distinguishing these from non-food-oriented chemical strategies. The review further highlights novel applications of modified sodium alginate in areas including food packaging, functional delivery systems, drug release, tissue engineering, and environmental remediation (heavy metal and dye removal). Overall, this work provides a comprehensive perspective linking modification pathways to food-relevant applications and clarifies how chemical tailoring of alginate contributes to the design of safe, sustainable, and high-performance bio-based materials.

## 1. Introduction

Sodium alginate (SA) is a linear copolymer composed of β-D-mannuronic acid (M) and α-L-guluronic acid (G) units [1]. Its unique egg-box gel structure and M/G ratio-dependent properties make it a popular study topic in natural polymer materials. With the growing emphasis on green chemistry and sustainable development, SA has received much attention in food engineering, biomedicine, and environmental remediation due to its wide availability, high biocompatibility, and strong modifiability [2]. Furthermore, SA is officially recognized as a safe and approved additive by major regulatory authorities. In the European Union, it is listed as food additive E401 under Regulation (EC) No 1333/2008 and specified in Commission Regulation (EU) No 231/2012 [3,4]. In the United States, sodium alginate is classified as a “Generally Recognized as Safe” (GRAS) substance under 21 CFR § 184.1724 [5] by the Food and Drug Administration (FDA), where it is permitted for use as a stabilizer, thickener, and emulsifier. These regulatory approvals further validate its broad and safe applications in food, pharmaceutical, and biomedical fields. It should be noted that the chemical tailoring discussed herein is directed to the design of edible and food-contact materials, rather than the authorization of new food additives per se. This clarification ensures that the review focuses on food-safe modification routes and materials relevant to food science, rather than legislative approval processes.

Despite its advantages, SA often exhibits inherent limitations in mechanical strength, environmental stability, bioactivity, and controlled release [6]. To overcome these shortcomings, three major modification approaches—chemical, physical, and enzymatic—have been developed. Previous reviews have typically addressed only individual modification pathways or specific properties, lacking a comprehensive and application-oriented perspective. This review, therefore, integrates current progress across all three modification domains, examining molecular mechanisms, process routes, and resulting functionalities. Particular emphasis is placed on food-compatible and green chemistries (e.g., phenolic crosslinking and enzyme-assisted coupling) that support the development of edible films, smart/active packaging, 3D-printed constructs, and functional food carriers, while stronger synthetic chemistries are discussed as forward-looking strategies for non-food fields.

**Figure 1:**
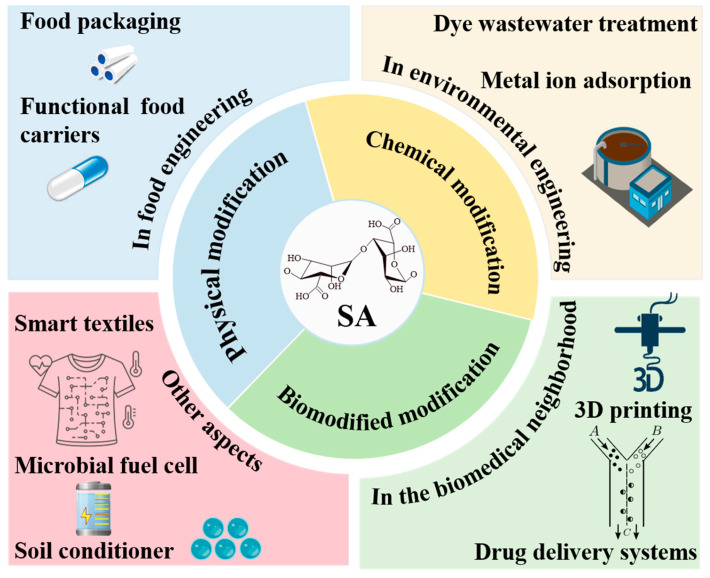
Conceptual roadmap illustrating the main modification strategies of sodium alginate (chemical, physical, and enzymatic) and their corresponding application fields. In the drug-delivery schematic, A and B represent two inlet channels introducing different drug formulations, while C denotes the outlet channel. The arrows indicate the flow direction during microfluidic-assisted mixing.

This synthesis establishes a coherent framework for understanding structure–property relationships and guides engineering of multifunctional, sustainable SA-based materials for food systems (see Figure 1).

## 2. Preparation of Sodium Alginate

**Figure 2:**
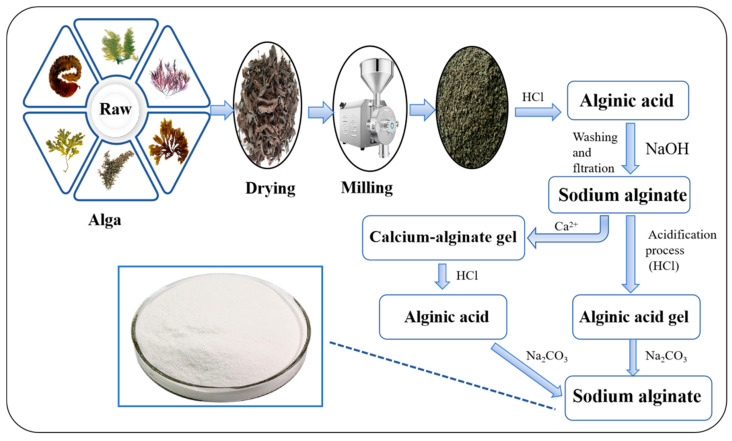
Schematic diagram of the traditional extraction of sodium alginate.

Alginate is a naturally abundant polysaccharide found in brown algae and plays a key structural role. The primary production sources include Macrocystis pyrifera (giant kelp), Laminaria digitata (oarweed), Laminaria japonica (kombu), Ascophyllum nodosum (knotted wrack), and other macroalgae [7]. The traditional extraction process [8] of SA from brown algae generally involves several key steps, including acid pretreatment [9], alkaline extraction [10], filtration, and drying [11], as illustrated in Figure 2. In this process, hydrochloric acid pretreatment helps remove impurities and enhance viscosity, followed by sodium carbonate extraction and purification to obtain high-purity SA. Recently, ultrasound-assisted extraction [12] has been introduced as an efficient alternative method to improve extraction efficiency, shorten processing time, and maintain the structural integrity of polysaccharides. Youssouf et al. [13] developed an ultrasound-assisted extraction (UAE) [14,15,16] procedure for SA preparation from brown algae (*Sargassum binderi* and *Turbinaria ornata*), while simultaneously enabling the extraction of carrageenan from red algae (*Kappaphycus alvarezii* and *Euchema denticulatum*). The UAE technique can effectively maintain the conformational integrity of target polysaccharides and significantly improves the extraction efficiency, providing a new technological pathway for algal polysaccharides production. Recently, Ummat et al. [17] used ultrasound-assisted sodium bicarbonate [18,19] to extract alginate from solid-phase by-products obtained after conventional extraction of fucoidan from brown algae [20], resulting in improved extraction yields.

## 3. Physicochemical Properties of Sodium Alginate

### 3.1. Chemical Structure and Molecular Weight

**Figure 3:**
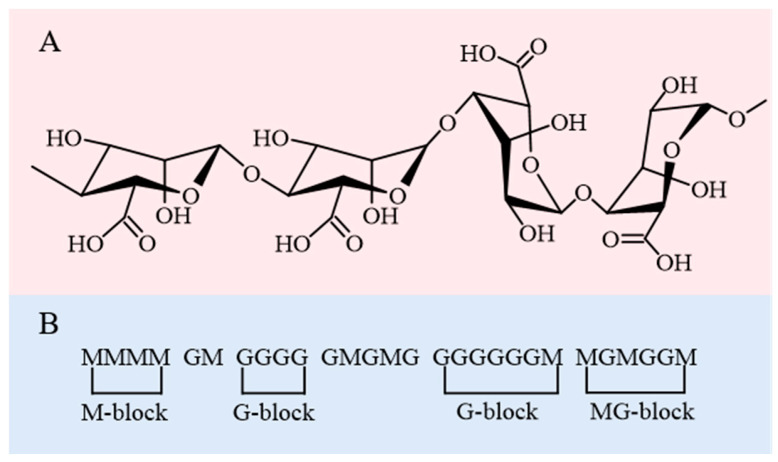
Chain conformation (**A**) and block distribution (**B**) of sodium alginate, where M and G represent β-D-mannuronic acid and α-L-guluronic acid residues, respectively.

SA is a natural linear copolymer obtained from seaweed cell walls, consisting of (1→4)-linked β-D-mannuronic acid (M unit with a ^4^C_1_ ring conformation) and α-L-guluronic acid (G unit with a ^1^C_4_ ring conformation) residues [21]. Its structure is characterized by homopolymeric blocks (continuous M or G sequences) and heteropolymeric blocks (alternating M and G units), as shown in Figure 3A [22]. More specifically, SA exhibits an irregular block architecture with varying proportions of MG, GM, GG, and MM blocks, as illustrated in Figure 3B [23,24]. SA from various sources varies in the M/G ratio, the length of the polymer chains, and the degree of blockiness or randomness in the distribution of M and G residues, which predominantly determine its structural characteristics as well as physicochemical properties. The proportion and distribution of GulA and ManA units vary with seaweed species. The relative abundance and distribution of GulA and ManA units, as well as their chemical modification, can significantly influence the physicochemical properties of SA. SA enriched with GulA units shows a rigid molecular structure, whereas SA rich in ManA units possesses a flexible structure [25]. The ratio of MM, GG and MG blocks determines the physical properties of SA [8]. For example, SA with a high G content has a higher gelling capacity, whereas SA with a high M level exhibits a higher viscosity [26]. The assessment of the M/G ratio is also important since a high M/G ratio causes SA to form an elastic gel, whereas a low M/G ratio produces brittle gels [27,28]. Only the G-block of SA is thought to contribute to the formation of hydrogels by intermolecular cross-linking with divalent cations (e.g., Ca^2+^) [29].

The molecular weight of SA varies considerably, as it is not genetically encoded but enzymatically synthesized by alginate polymerases and can undergo partial depolymerization during extraction and purification [27]. Consequently, the molecular weight of SA is conventionally expressed as an average value, typically represented by the number-average (Mn) and weight-average (Mw) molecular weights [28]. Reported Mw values span a broad range—approximately 60,000 to 700,000 g/mol for commercial alginates [30], and in some cases reaching up to ~986,000 g/mol depending on algal species and extraction conditions [31]. Commercial-grade materials from suppliers such as Sigma-Aldrich (Product PHR1471) are generally specified within the 12,000–40,000 g/mol range, reflecting controlled depolymerization used to standardize viscosity for analytical and industrial purposes [32]. The molecular weight distribution of SA is influenced by several factors, including biological source, extraction chemistry (acidic vs. alkaline), temperature, oxidative conditions, and mechanical shear during processing [33]. The polydispersity index (PDI = Mw/Mn), which characterizes the breadth of chain-length distribution, typically falls between 1.5 and 3.0, though values up to 6 have been reported for heterogeneous or partially degraded alginate preparations [34]. Collectively, these findings indicate that the molecular weight of SA should be regarded as a variable distribution dependent on biological origin and processing history, rather than a single fixed value.

### 3.2. Solubility

The solubility of SA in aqueous media is influenced by several factors, including solution pH, the presence of co-solvents, ionic strength, gel-promoting ions, and the intrinsic structure of the biopolymer. Dissolution of SA requires a pH above a critical threshold necessary for the deprotonation of its carboxyl groups [35]. The solubility of SA is fundamentally determined by its molecular structure and the ionization state of the carboxyl group in the polymer backbone. When the carboxyl groups are protonated to form alginic acid, the polymer exhibits minimal solubility and becomes insoluble in most solvents, including water. In contrast, salt forms of SA are water-soluble but remain insoluble in organic solvents, such as alcohol, hydroalcoholic solutions containing at least 30% alcohol, chloroform, and ether. An exception is the tetrabutylammonium (TBA) salt of SA, which is soluble in water, ethylene glycol, and polar aprotic solvents containing tetrabutylammonium fluoride (TBAF) [36]. Under acidic conditions, the solubility of SA is significantly reduced due to limited hydration capacity [22]. SA dissolves slowly in cold water, forming viscous solutions. Furthermore, the ability of alginate to form hydrogels via cross-linking with divalent ions such as Ca^2+^, Ba^2+^, and Zn^2+^ considerably influences its solubility behavior [27].

### 3.3. Gel Formation Ability

**Figure 4:**
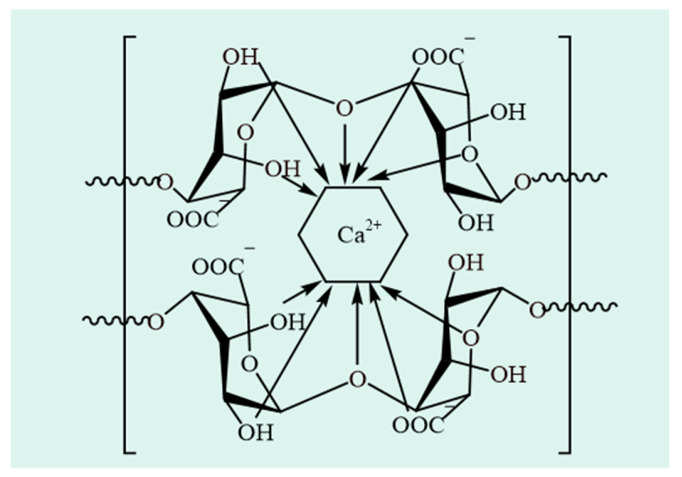
“Egg-box” gel structure of sodium alginate, illustrating the coordination of Ca^2+^ ions within G-block regions.

Gelation of SA can be induced by cations such as H^+^, Ca^2+^, Cu^2+^, Ba^2+^ [37], primarily through electrostatic interactions between negatively charged carboxyl groups on the polymer chain and positively charged cations, leading to the formation of ionic cross-links and resultant polyelectrolyte complexes [38]. Ca^2+^-induced gelation represents one of the most important functional properties of SA [39]. This process is facilitated by ion exchange between sodium ions associated with G units of SA and calcium ions, which promotes the aggregation of G-blocks into a characteristic egg-box gel structure (Figure 4) [40]. There are two key methods of forming calcium ionic crosslinked hydrogels: internal gelation and external gelation. Internal gelation involves adding Ca^2+^ directly to the polymer dispersion and stirring for a specific duration to activate the ionic cross-linking reaction [41]. The properties of gel formed by internal gelation usually depend on the calcium salt used. For example, calcium chloride cross-links very rapidly with SA, forming a heterogeneous gel structure [42]. External gelation of Ca^2+^ usually involves immersing the pregel into a Ca^2+^ solution, where the Ca^2+^ diffuses inward to form the gel and replaces the Na^+^ in the SA structure [43]. External gelation enables rapid gel formation (within seconds) without disrupting the continuity of the pre-gel structure, and the crosslink density of the final gel network correlates with the Ca^2+^ concentration [44]. Alginates also form gels with a variety of other mono-, di-, or trivalent cations. The gelation mechanisms [45] for these cations often differ from Ca^2+^-induced gelation, resulting in gels with diverse structural characteristics [46] and potential applications [47].

### 3.4. Biocompatibility

Alginate is a biocompatible and biodegradable polymer whose properties vary significantly based on its G/M ratio and overall chemical composition [48]. The immunogenicity of SA is influenced by its structural features and the presence of specific functional groups. For example, alginates rich in M units are more immunogenic and effective in triggering the generation of cytokines compared to those with a high G content [49]. Therefore, structural modifications represent a viable strategy to modulate its immunogenic potential [50]. SA hydrogel [51] is widely used in the biomedical field due to its inherent biocompatibility and non-toxicity. For instance, a novel hydrogel loaded with Ag-Metal–organic frameworks has been developed to enhance its antibacterial efficacy [52]. SA-based hydrogels demonstrate adequate biological safety profiles, making them suitable for wound healing applications [53]. Moreover, these hydrogels can be tailored to release drugs in a controlled manner, making them ideal for controlled drug delivery systems [27].

## 4. Modification Methods of Sodium Alginate

In recent years, research on SA modification has shifted from single property optimization toward multifunctional synergistic design. To meet diverse material requirements in different fields, researchers have developed proposed multidimensional modification strategies, typically classified into three major approaches: chemical modification, physical modification, and biological modification.

### 4.1. Chemical Modification

SA is widely used due to its superior biocompatibility and processability. Researchers have developed various chemical modification methods, including esterification, oxidation, sulfation, Ugi reaction, aldehyde cross-linking, phosphorylation, amidation, and grafting, to enhance its functional attributes. These methods precisely control the molecular structure of SA, imparting hydrophobicity, enhancing adsorption/binding capacity, introducing bioactivity, optimizing drug delivery profiles, improving stability, regulating dissolution/mineralization properties, enhancing processability, and conferring smart responsive characteristics to the material. As a result, the potential of SA in the design and application of functional materials has been significantly expanded. To align the discussion with food science, we classify chemical modifications into two practical categories: (i) food-compatible or green chemistries potentially suitable for edible/food-contact uses (e.g., phenolic crosslinking with tannic/cinnamic acids, genipin crosslinking, enzyme-assisted coupling), and (ii) non-food chemistries (e.g., strong sulfation or periodate oxidation, multi-component Ugi reactions, certain vinyl-graft routes) that are mainly forward-looking material strategies. Throughout this section and the Applications part (Section 5), we explicitly indicate when a given chemistry is relevant to food systems versus non-food domains.

#### 4.1.1. Esterification

**Figure 5:**
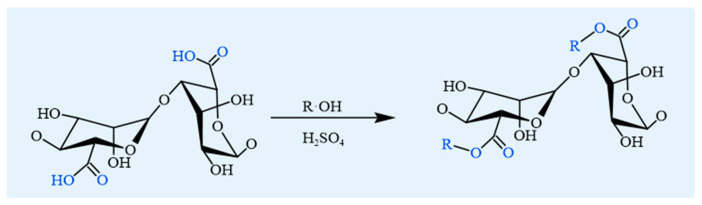
Esterification of sodium alginate: formation of ester linkages between hydroxyl and acyl groups.

Esterification of SA typically involves an acid-catalyzed dehydration and condensation reaction between carboxyl and hydroxyl groups to form ester bonds (Figure 5). This reaction also provides a straightforward method for attaching alkyl groups to the main chain of SA [54]. Esterification procedures can be categorized into two methods: (i) surface modification of alginate products. For instance, esterification of maleic anhydride on the Ca^2+^–alginate hydrogel beads has been shown to improve their oil adsorption capacity [23]; (ii) molecular modification prior to product preparation. Esterification can improve the mechanical properties of SA by introducing ester groups into its polymer chains. This modification enhances both rigidity and tensile strength, making it more suitable for applications requiring higher mechanical stability, such as hydrogels [55]. Specifically, maleic anhydride esterification significantly increases the hydrophobicity and oil adsorption capacity of SA, which forms a hydrogel that can efficiently capture oil droplets in water, suitable for oil cleanup and wastewater treatment [56]. Similarly, thioacetic acid esterification considerably improves the mechanical properties and bioadhesion of SA, showing promising potential for biomedical applications such as drug delivery and wound healing [57].

Food relevance: Certain esterification reactions of sodium alginate can be food-compatible when employing food-grade or naturally derived acids (e.g., fatty, phenolic, or cinnamic acids). These mild routes are useful for tuning hydrophobicity and barrier properties in edible films and food-contact materials, whereas strong acid-catalyzed reactions remain non-food.

#### 4.1.2. Oxidization

**Figure 6:**
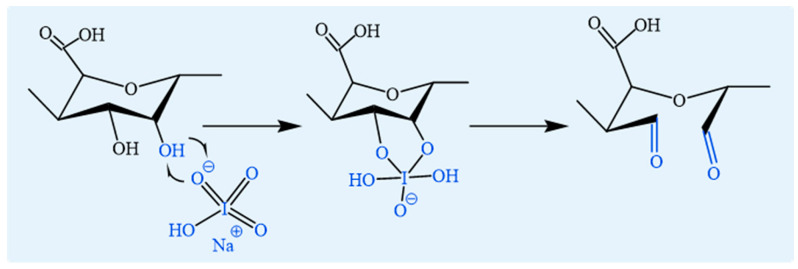
Oxidation of alginate chains via periodate cleavage of vicinal diols to generate dialdehyde sites.

SA can be oxidized by sodium periodate to introduce aldehyde groups (Figure 6). This reaction selectively targets the C-2 and C-3 positions of the hyaluronic acid units, converting them into aldehyde groups. This modification enhances the binding affinity of alginate toward heavy metal ions [58]. Furthermore, the process of oxidation significantly alters the molecular structure and properties of alginate, including its aldehyde content and molecular weight [23]. The gravimetric molar mass of alginate decreases rapidly with increasing degree of oxidation, even at low levels (e.g., 5 mol%) [58]. Notably, when the molar ratio of sodium periodate to alginate is held constant, the amount of aldehyde groups produced on the oxidized polymer backbone increases with increasing alginate concentration. Concurrently, the molecular weight decreases as alginate concentration increases, probably due to enhanced chain breakage. This occurs because higher molecular collision frequencies in concentrated solutions accelerate oxidation of adjacent hyaluronic acids within chains [59]. To minimize the possibility of side reactions, the oxidation procedure should be performed in complete darkness.

Food relevance: Mild oxidation using controlled periodate or enzymatic routes can be applied for structural modulation in edible coatings and hydrogels, but high-degree oxidation is mainly non-food and used for biomedical/environmental purposes.

#### 4.1.3. Sulfation

**Figure 7:**
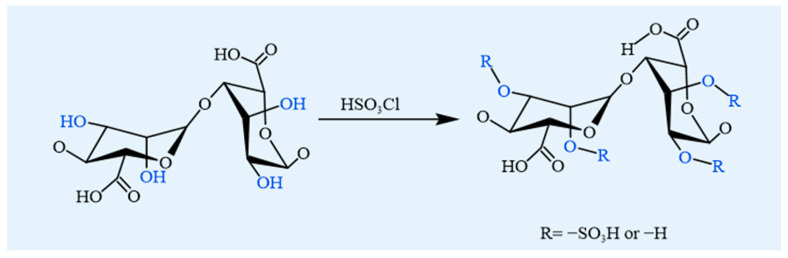
Sulfation of alginate through substitution of hydroxyl groups with sulfate esters (–OSO_3_^−^), enhancing hydrophilicity.

Sulfation is a prominent modification strategy for polysaccharides in biomedical research due to its broad range of bioactivity, including anticoagulant, anticancer, antimicrobial, antiviral, and immunomodulatory effects [60]. This chemical process involves introducing sulfate groups onto the hydroxyl residues of alginate, resulting in the formation of sulfated alginate derivatives (Figure 7). Common methods involve reacting alginate with chlorosulfonic acid or 1,3-benzene sulfonyl chloride in formamide [23]. Among various sulfating agents used for sulfation of alginate (e.g., sulfuric acid, chlorosulfonic acid, sulfuryl chloride, sulfamic acid, and sulfur trioxide), chlorosulfonic acid is particularly suitable for alginate functionalization due to its reaction controllability and reproducibility [61]. The introduction of sulfate groups induces conformational changes in the three-dimensional structure of the polysaccharide chain, thereby enabling these polysaccharides with weak or no bioactivity to enhance or acquire some sulfate group-related activities [62]. Sulfated polysaccharides have demonstrated a wide range of biological activities, such as anticoagulant, anti-inflammatory, antiviral, and immunomodulatory properties, making them beneficial for biomedicine, functional food, and biomaterial applications [63].

Food relevance: Sulfation commonly involves non-food reagents (e.g., chlorosulfonic acid) and thus remains non-food; its relevance is confined to biomedical studies rather than direct food-contact applications.

#### 4.1.4. Ugi Reaction

**Figure 8:**
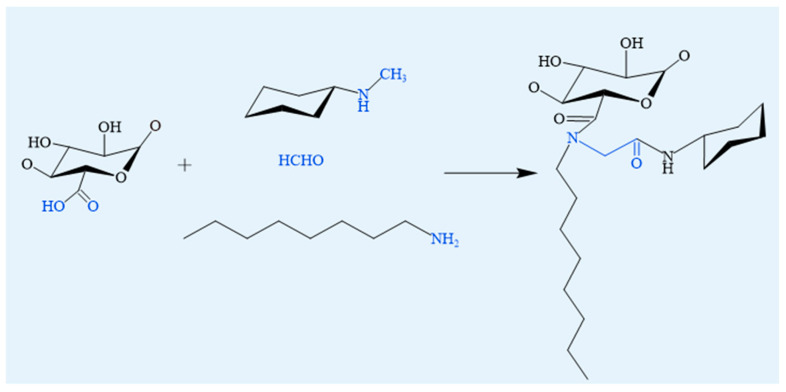
Ugi multi-component reaction forming amide linkages between amino, aldehyde, carboxyl, and isocyanide groups.

The Ugi reaction is a four-component coupling process involving a carboxylic acid, a carbonyl compound, an amine, and an isocyanide. This reaction allows the efficient synthesis of amino acid and peptide derivative libraries [64]. All reaction components are in a dynamic equilibrium through several intermediates, until an irreversible intramolecular 1,4-O→N acyl transfer yields an N-acylamino acid amide [65] (Figure 8). Amphiphilic alginate derivatives with tunable organic solubility and thermal properties have been successfully synthesized via the Ugi multicomponent reaction [66]. This approach incorporates hydrophobic components, such as octylamine and oleylamine, into the SA backbone, significantly enhancing its hydrophobicity and improving its self-assembly behavior [67]. The resultant amphiphilic SA derivatives demonstrate enhanced ability to encapsulate hydrophobic drugs and exhibit improved drug release profiles, highlighting their potential for hydrophobic drug delivery systems [68].

Food relevance: Because the Ugi reaction uses isocyanides and organic solvents, it is not food-compatible and is considered a non-food modification, summarized here only for its structural design value.

#### 4.1.5. Aldehyde Cross-Linking

**Figure 9:**
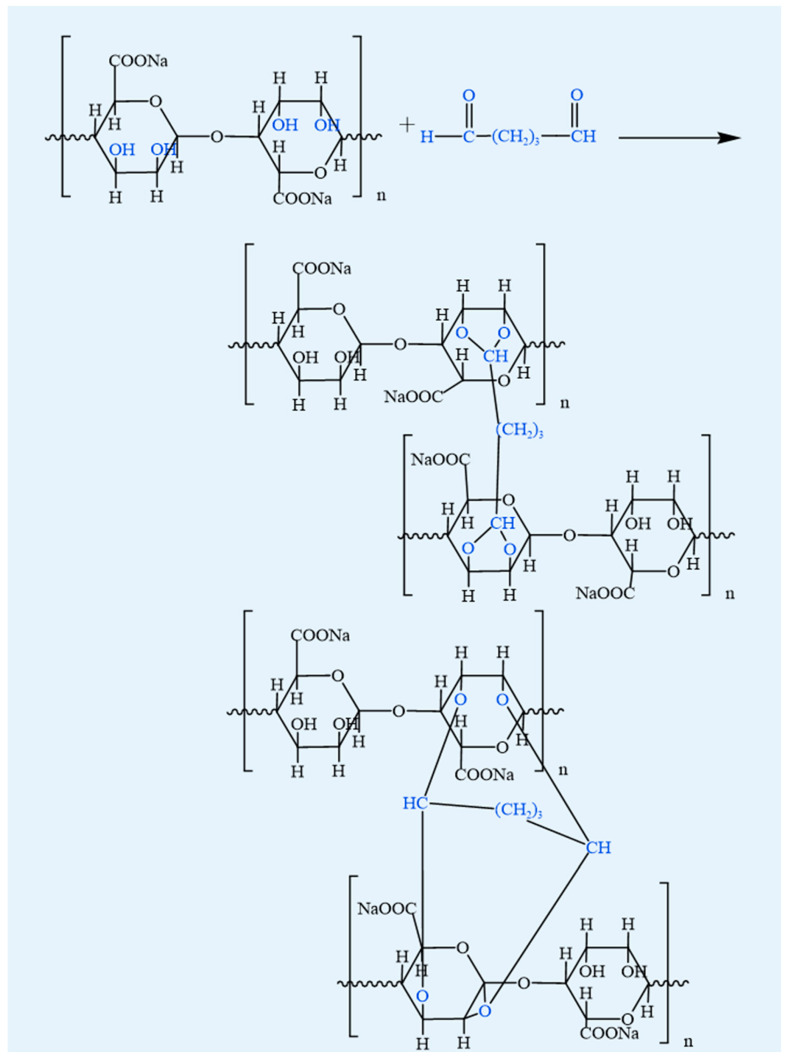
Aldehyde cross-linking through Schiff-base formation between aldehyde and hydroxyl or amino groups.

Aldehyde cross-linking forms stable covalent linkages between aldehyde and amino groups, thereby enhancing the structural stability of biomolecules [69] (Figure 9). In collagen, lysyl oxidase generates crucial intermolecular cross-links through telopeptide lysine aldehydes and telopeptide hydroxylysine aldehydes [70,71]. However, in hydrogels, aldehyde cross-linking occurs via both covalent and Schiff-base pathways. Covalent cross-linking involves the formation of stable bonds between aldehyde and amino groups, whereas Schiff base cross-linking induces the formation of reversible linkages [72]. Aldehyde cross-linking offers an alternative strategy to reinforce the stability and functionality of alginate-based materials [73]. For instance, dialdehyde starch has been employed to crosslink gelatin and SA hydrogels, enabling precise modulation of their mechanical properties. This approach significantly improves the stability and functionality of the hydrogels [74].

Food relevance: Naturally derived aldehyde donors (e.g., genipin or reducing sugars) enable safe crosslinking for food-contact or edible materials, whereas strong aldehydes like glutaraldehyde are non-food and restricted to biomedical/industrial uses.

#### 4.1.6. Phosphorylation

**Figure 10:**
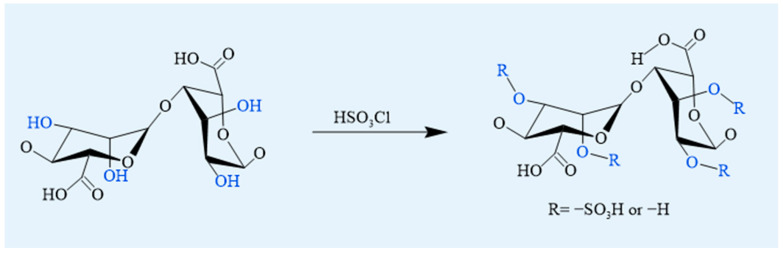
Phosphorylation introducing phosphate groups that improve metal chelation and biological functionality.

Phosphorylated alginates are commonly used to promote nucleation and growth of hydroxyapatite (HAP). Phosphorylation is typically carried out using a mixture of urea and phosphoric acid (Figure 10) [35]. Coleman et al. [75] successfully functionalized alginate hydroxyl groups through heterogeneous urea/phosphate reactions. Multidimensional NMR studies indicate that phosphorylation preferentially targets the C3 equatorial carbon of mannuronate (M) residues, although the reactivity of guluronate (G) units remains unclear [66]. The modification can considerably alter the physicochemical properties of alginate, including its solubility, viscosity, and gelation behavior, while also enhancing its bioavailability [76] via improved biomolecular interactions [30]. Wang et al. [77] established a straightforward method for fabricating macroporous alginate hydrogels, which involves dissolving phosphorylated SA in water and using Ca^2+^ released from acid-induced CaCO_3_ dissolution to trigger gelation, during which CO_2_ bubble formation creates pores. The resulting hydrogels display dynamic and reversible viscoelasticity, making them highly suitable for applications requiring tunable mechanical performance [78].

Food relevance: Due to the use of non-food reagents and migration concerns, phosphorylated alginates have limited direct food applicability and are primarily used in biomedical and nutrient-delivery models.

#### 4.1.7. Amidation

**Figure 11:**
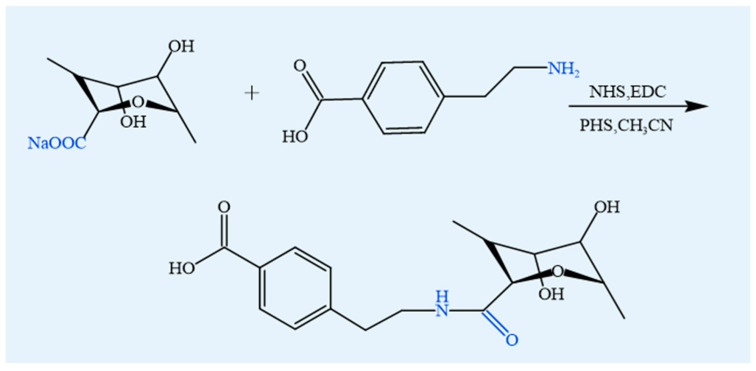
Amidation reaction producing amide bonds between carboxyl and amino groups via carbodiimide coupling.

Amidation is a reaction that involves the formation of amide bonds between carboxyl groups and amine groups, facilitating the incorporation of hydrophobic tails into alginate and other polysaccharides (Figure 11). This modification enhances their ability to self-assemble into micelles and vesicles in aqueous environments [79]. Using 4-(4,6-dimethoxy-1,3,5-triazin-2-yl)-4-methylmorpholinium chloride (DMTMM) as a condensing agent, alginate can be efficiently functionalized with various amines such as furanamine, adipic acid dihydrazide, amino acids, and peptides. The reaction proceeds through a pH-dependent mechanism, with optimal efficiency observed at pH 5–6, leading to amide bond formation between the carboxyl groups of alginate and the amino groups of the modifiers [80]. DMTMM-mediated amidation with amino acids, including alanine, leucine, and serine, significantly enhances the enzymatic resistance of alginate oligosaccharides against alginate lyase [81]. Moreover, amidation disrupts intramolecular hydrogen bonding, resulting in reduced surface tension and conductivity of electrospun solutions, while enhancing spinnability and molecular flexibility [82]. The modified alginate also exhibits greater binding affinity and selectivity toward both anionic and cationic dyes, underscoring its potential as an effective adsorbent material [83].

Food relevance: Amidation can be food-compatible when the amine donors are natural biomolecules (e.g., amino acids, peptides), providing enhanced digestibility and biocompatibility for functional food carriers; synthetic amines remain non-food.

#### 4.1.8. Graft Modification

**Figure 12:**
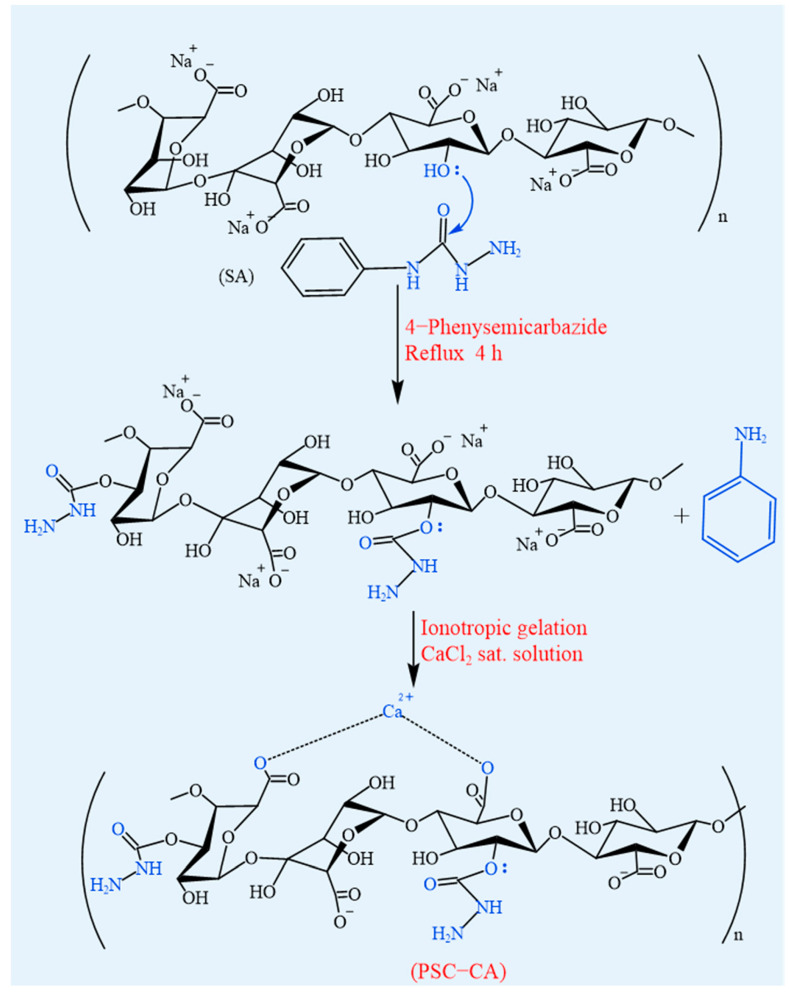
Grafted calcium alginate (PSC–CA) hydrogel beads functionalized with amino-carbamate moieties for enhanced strength and adsorption.

The limitations of SA in terms of its strong hydrophilicity, poor mechanical properties, and limited stability have driven significant interest in graft modification as a means to enhance its functionality [84]. The grafting technique is widely used to modify SA that generates active sites (e.g., free radicals or functional groups) on the polymer backbone [2]. The introduction of the desired components can effectively improve mechanical strength, thermal responsiveness, and compatibility of SA, while preserving its natural properties and biocompatibility [85]. Grafting methods are mainly classified into free radical grafting and ionic grafting. In free radical grafting, the active site exists in the form of a functional group or free radical, triggering the reaction of the monomer with the polymer backbone. In contrast, ionic grafting utilizes ionic active sites to attach monomers through ionic interactions. For example, Sand et al. [86] modified alginate by grafting N-vinyl-2-pyrrolidone via free radical initiation, resulting in improved swelling behavior, metal-ion adsorption, and flocculation properties. Similarly, free radical-induced grafting has been employed to synthesize diethylmalonic acid-grafted chitosan and alginate-g-poly (1-carboxy-4-acrylamidobenzenesulfonamide) copolymer [87,88], whose freeze-dried forms show great potential in water treatment. Shehzad et al. [89] developed calcium carbamate-partially grafted alginate hydrogel beads by reacting SA with calcium chloride and 4-phenylcarbamyl urea (Figure 12). These beads enable efficient and economical recovery of the silver ion (Ag^+^) from aqueous solutions. Ionic cross-linking is a major strategy for functionalizing grafted sodium alginate (NaAlg). For instance, Fe^3⁺^-crosslinked acrylamide (AAm)-grafted poly (vinyl alcohol) (PVA)/NaAlg microspheres achieved controlled release of the anticancer drug 5-fluorouracil (5-FU), with release behavior modulated by NaAlg content [90]. Similarly, Sr^2+^-crosslinked alginate hydrogels have been shown to significantly enhance mechanical strength and bioactivity, supporting their potential for biomedical applications [91]. Additionally, ionically crosslinked AAm-grafted PVA/NaAlg/sodium carboxymethylcellulose pH-sensitive microspheres have been developed for the delivery of donepezil hydrochloride, a drug used in Alzheimer’s disease treatment [92]. These examples demonstrate how ionic crosslinking synergized with grafting (e.g., Fe^3^⁺, Sr^2^⁺) enhances drug-controlled release capability, mechanical properties, and environmental responsiveness in SA-based materials.

Food relevance: Graft modification can also be adapted for food-contact or edible applications when natural or biocompatible monomers—such as polysaccharides, polyphenols, or amino acids—are employed, leading to functional coatings and smart packaging materials. In contrast, grafting with synthetic vinyl or acrylate monomers remains outside the scope of food use and is primarily suited for biomedical and environmental applications.

Overall, not all chemical routes are directly applicable to food systems; however, distinguishing food-compatible from non-food chemistries clarifies the relevance of alginate functionalization to food science. Mild esterification, amino-acid amidation, and natural crosslinking (e.g., phenolic or genipin routes) represent safe and effective strategies to enhance edible and food-contact materials, whereas stronger or synthetic chemical reactions are retained as forward-looking, non-food-oriented approaches.

### 4.2. Physical Modification

#### 4.2.1. Composite Modification

Alginate-based composite material systems have attracted growing attention in functional materials research due to their high tunability and wide range of performance control. By employing multi-component synergistic strategies, the benefits of different materials can effectively be integrated, overcoming the limitations of single alginate systems in terms of mechanical strength and functional responsiveness. Current research in this area focuses primarily on three technical pathways: polymer blending, inorganic materials composites, and nanomaterial hybridization. The latter refers to the incorporation of nanostructured components (e.g., graphene, nanoclay, carbon nanotubes, metal or oxide nanoparticles) into the alginate network to generate synergistic interfacial effects, such as enhanced mechanical strength, conductivity, or barrier performance. Common fabrication techniques, such as homogeneous blending and in situ synthesis, frequently utilize the sol–gel phase transition of SA to immobilize functional components within three-dimensional network structures. This achieves synergistic enhancement of mechanical properties, biocompatibility, and environmental responsiveness. These multi-scale composite strategies provide a valuable approach for developing advanced functional materials like smart drug carriers and flexible sensors.

##### Polymer Material Composite

**Figure 13:**
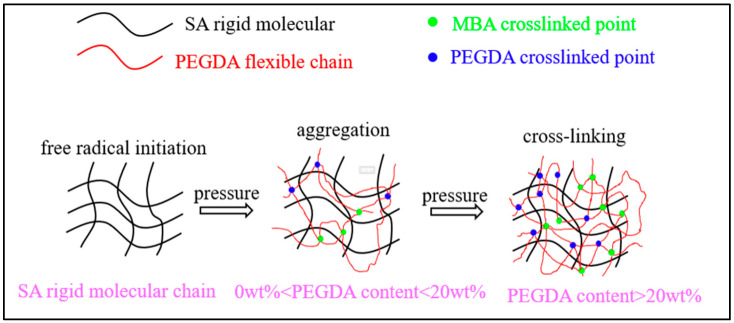
Simplified schematic illustration of the structural evolution and rheological behavior of SA/PEGDA systems under increasing PEGDA content.

Modification of SA with polyethylene glycol diacrylate (PEGDA) has been widely investigated to improve material performance, showing significant potential in biomedical applications. Incorporation of PEGDA chains effectively reduces viscosity and enhances rheological behavior of the alginate solution, improving spinning performance and enabling the fabrication of diverse dual-network structures, such as microspheres and thin films, via modulated molding processes [27,93]. Further, Zhou et al. [94] have developed a “one-pot” synthesis method to produce high-strength semi-interpenetrating SA/PEGDA dual-network fibers, and their rheological schematic diagram is shown in Figure 13. For wound repair, integrated SA/PEGDA hydrogel systems (integrated polyethylene glycol/alginate-based hydrogel) leverage structural designs for functional synergy [95]. In tissue engineering scaffold design, sodium alginate (ALG)/PEGDA composite systems utilize “primary-secondary” dual network structures (PABC scaffolds) to accomplish multifunctional integration and enhanced performance [96]. The system combines the bioactivity and degradability of SA (by ionic crosslinking) with the mechanical stability and antioxidant function of PEGDA (by covalent crosslinking). This integration not only optimizes the rheological properties and processing adaptability of the material but also achieves multi-functional integration of antimicrobial, pro-restorative, and self-restorative functions through the spatial structure modulation.

Chitosan (CS) and its derivatives, as polycationic backbones, interact electrostatically with the polyanionic alginate, forming stable polyelectrolyte complexes [97]. The literature shows that SA/CS composites demonstrate remarkable synergistic effects in biomedical materials through polyelectrolyte complexation, dynamic cross-linking, and multi-scale structural design. The SA/CS composite systems provide many functional advantages in wound repair materials due to the synergistic effect of polyelectrolyte self-assembly and cross-linking, which endows the material with optimized mechanical strength (adapted to the dynamic mechanical environment of the wound) and interconnected porous architecture to provide physical support for cell migration and neovascularization [98]. Guan et al. [99] successfully developed a CS-SA (GTA0.3) gel system with a semi-decomposed chain network structure by glutaraldehyde (GTA)-mediated polyelectrolyte composite. Among them, the moderate cross-linking of GTA weakened the rigidity of the conventional dense polyionic network of CS-SA, forming a loose and porous amorphous structure. Additionally, Li et al. [100] developed biomedical composites with a bilayer porous structure by synergizing SA with chitosan-based materials. Both of them successfully fabricated an ordered laminar porous skeleton using high-speed homogeneous foaming and two-step freeze-drying. Building on this foundation, Zhao et al. [101] developed a composite system of carboxymethyl chitosan (CMCS) and sodium alginate (OSA). Using dynamic chemical bonding and in situ metal nanoparticle integration, they fabricated functional electroactive hydrogels (OSA/CMCS/AgNPs) without external conductive additives, establishing an innovative paradigm for smart biomaterial design. Song et al. [102] further demonstrated that this system exhibited outstanding hemostatic performance due to polyelectrolyte synergy and multiscale architecture. The flexible SA chains and the stiff chains of CMCS in the composite system synergize to enhance the mechanical properties, preventing the secondary damage caused by the insufficient mechanical strength of traditional hemostatic materials. In summary, SA/CS interpenetrating networks formed by electrostatic self-assembly not only optimize the mechanical properties and porous structure of the materials, but also confer antimicrobial, pro-restorative, adsorptive, and electroactive functions through the synergistic effect of functional groups (amino, carboxyl, etc.). These structurally innovative systems, driven by intermolecular interactions, when combined with freeze-drying and in situ nanoparticle integration, provide multifunctional platforms for the development of smart hemostatic materials, dynamically responsive wound dressings, and environmental remediation materials, significantly expanding the boundaries of biomedical applications of natural polysaccharide-based composite systems.

Polyvinyl alcohol (PVA), a water-soluble polymer with pronounced film-forming properties, has emerged as a promising material for combinations with SA due to its low density, processability, and microbial compatibility [103]. SA and PVA demonstrate multidimensional synergistic benefits in biomedical and environmental engineering via physical cross-linking, dynamic bonding, and functional composite strategies. These polymers form three-dimensional networks [104] and antibacterial composite gel beads (C/PVA/SA) [105] through physical cross-linking. On the other hand, the flexible cross-linking network of SA/PVA protects microorganisms from environmental stresses (e.g., extreme pH or temperature), strengthens material robustness, and provides a highly efficient carrier design strategy for pesticide contamination bioremediation. Furthermore, PVA, as a flexible reinforcing agent, significantly improves the mechanical strength and deformation resistance of the material through the formation of a hydrogen bonding between hydroxyl and carboxyl groups of SA. SA and PVA can also be used to develop dynamically reversible hydrogels [106] and physically cross-linked scaffolds [107], where the anionic properties of SA and hydrogen bonding of PVA synergistically create three-dimensional porous networks. These confer the scaffolds with good hydrophilicity and tunable degradation, and also endow the composite hydrogels with robust mechanical toughness and self-recovery properties. In summary, the three-dimensional porous structure fabricated by SA and PVA combines the biological activity and degradability of SA with the mechanical stability of PVA. Functional integration of biochar, nanosilver, or conductive materials further imparts synergistic adsorption–degradation, broad-spectrum antibacterial activity, and intelligent sensing properties.

##### Inorganic Material Composite

SA and calcium carbonate (CaCO_3_) exhibit strong potential for innovation in environmental remediation, smart materials, and flexible electronics through dynamic ionic cross-linking (Ca^2+^-COO-) and multifunctional composite strategies. For example, they can function as anticorrosive pigments [108] or enhance the mechanical and electrochemical performance of smart hydrogel [109]. The carboxyl groups of SA interact with Ca^2+^ released from CaCO_3_ to form dynamic networks, which improve the densification and corrosion inhibition of the coating, stabilize the dispersion of pigment particles, and endow hydrogels with high tensile strength and environmental adaptability. Fu et al. [110] fabricated composite hydrogel beads by incorporating CaCO_3_ and bentonite (Be) into a carboxymethyl cellulose (CMC)/SA matrix. In this system, carboxyl groups from SA dynamically cross-linked with Ca^2+^, and when combined with the lamellar structure of Be, produced a homogeneous, dense, and porous surface. Fourier transform infrared (FTIR) and X-ray diffraction (XRD) analyses confirmed the stable embedding of CaCO_3_/Be.

Composite systems constructed by organic-inorganic hybridization strategies between SA and silica (SiO_2_) exhibit unique synergistic advantages in functional materials. Organic-inorganic interpenetrating polymer networks (IPNs) can be constructed by in situ hydrolytic condensation of SA and SiO_2_, typically using tetraethoxysilane (TEOS) as a precursor. Molecular-level interpenetration occurs through hydrogen bonding and electrostatic interactions between carboxyl groups of SA and silicone hydroxyl groups (Si-OH), with FT-IR confirming the chemical compatibility between the two phases [111]. SA and SiO_2_ are cross-linked with the assistance of CaCl_2_ to form CS/SA/SiO_2_ composites. Carboxyl groups of SA create an “egg-box” gel network with Ca^2+^, establishing a porous anionic skeleton and a nitrogen/nitrogen-containing/corporate network. The interfacial compatibility between SA and SiO_2_, achieved through hydrogen bonding and electrostatic interactions, forms folded surfaces and through-pore structures, which maintain the structural integrity of the material under thermal and aqueous stress, providing a composite design strategy for biomedical scaffolds or adsorbent materials that combines bioactivity and durability [106]. Based on the multiple interactions between SA and SiO_2_, researchers have constructed IPNs with multi-level pore structures. This dual-phase integration combines the bioactivity of SA with the rigidity of SiO_2_, improving both mechanical strength and environmental tolerance.

##### Nanomaterials

Nanomaterials [112] have distinct physicochemical properties compared to macroscopic materials due to their size effects, surface effects, and quantum effects. They typically possess high specific surface area, superb dispersibility, and tunable interfacial characteristics [113], demonstrating broad application potential in fields such as drug delivery, food packaging, antimicrobial materials, tissue engineering, and environmental remediation [114]. Recent advances in green synthesis have promoted the use of natural polymers as templates or stabilizers [115]. Among these, polysaccharides have garnered significant attention due to their intrinsic biocompatibility [116], renewability, and functionalization potential. SA serves as an effective biotemplate for the green synthesis and functional assembly of nanomaterials, utilizing its unique carboxylic acid moieties, porous network, and regulated ionic coordination [117]. For example, granular SA carriers formed via a high-shear wet granulation process can control the synthesis of ferrite oxide (NixFeyO_4_) nanoparticles. This method significantly reduces the high water consumption compared with conventional liquid-phase approaches, while simplifying the operational procedures and enhancing the process scalability [118]. SA hydrogel templates can direct the synthesis of Cu_2_O/Cu/@carbon heterostructures. The carboxyl groups of SA coordinate with metal ions to form a three-dimensional network, guiding the in situ assembly of Cu_2_O/Cu heterojunctions incorporated with reduced graphene oxide (rGO). The resulting hierarchical interface synergizes with the Schottky barrier of Cu_2_O/Cu through the electron conduction channel of rGO, significantly improving the efficiency of photogenerated carrier separation [119]. Moreover, SA can complex with copper-alumina nanoparticles to create ternary magnetoresponsive nanofluidic systems, where electrostatic interactions between anionic SA chains and nanoparticle surfaces control the viscoelastic and interfacial behavior [120]. Through solid–liquid exchange, hydrogel templates, or electrostatic dispersion, SA can effectively control the nucleation development and interfacial architecture of nanoparticles to augment material properties. Successful implementations in ferrite synthesis, heterojunction photocatalysts, and magnetic fluids illustrate the potential of SA in structure orientation and property optimization and provide a new way for the design and macro-preparation of environmentally friendly nanocomposites.

#### 4.2.2. Physical Processing Technique

##### Ultrasonication

**Figure 14:**
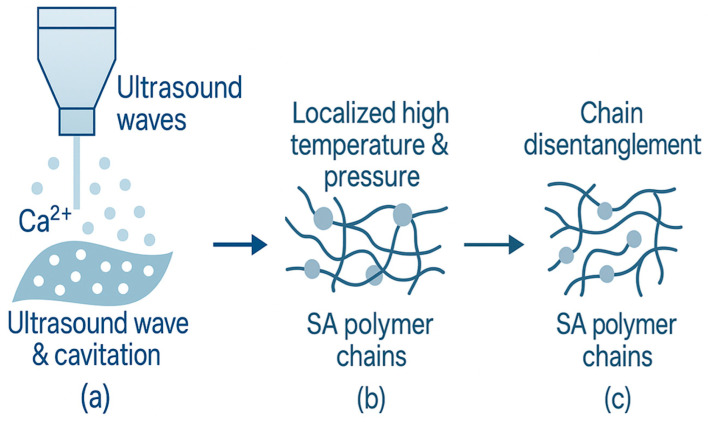
Schematic illustration of the ultrasonication-assisted modification of SA-based systems. (**a**) Ultrasound wave and cavitation process induced by Ca^2+^; (**b**) localized high temperature and pressure leading to rearrangement of SA polymer chains; (**c**) chain disentanglement and partial depolymerization of SA.

Ultrasonication is a non-thermal physical processing technique widely applied to modify the physicochemical and functional properties of food biopolymers such as SA. [121,122] As illustrated in Figure 14, ultrasound waves induce mechanical vibration and cavitation in the liquid medium, generating localized high temperature and pressure zones that enhance molecular motion, mixing, and mass transfer [123,124,125,126,127]. These cavitation effects disrupt polymer aggregates, promote chain disentanglement, and facilitate structural rearrangements, resulting in smoother, denser, and more homogeneous films [128,129,130,131]. Ultrasonication has been shown to significantly improve encapsulation efficiency and stability of bioactive compounds in SA-based films [132,133,134,135], and to modulate mechanical and barrier properties by tailoring polymer chain interactions. Moreover, ultrasound can induce conformational unfolding of proteins or enzymes (e.g., papain), exposing active sites and enhancing their binding affinity to SA matrices [136,137].

Overall, ultrasound-assisted processing provides a controllable and energy-efficient approach to enhance the structural and functional performance of SA-based materials.

##### Irradiance

**Figure 15:**
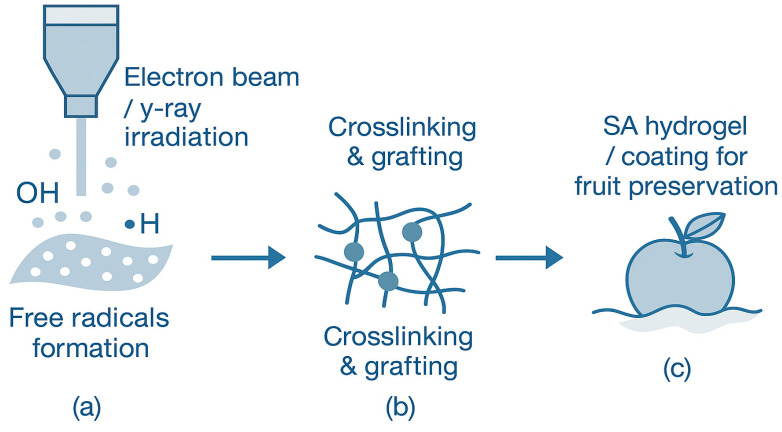
Schematic illustration of the irradiation-assisted modification of SA-based systems. (**a**) Formation of reactive free radicals through electron beam or γ-ray irradiation; (**b**) crosslinking and grafting reactions between SA molecular chains induced by the generated radicals; (**c**) formation of SA hydrogel or coating materials applied for fruit preservation and other functional uses.

Irradiation technology, including electron beam and γ-ray irradiation, provides an efficient and chemical-free route for modifying SA and expanding its applications in sustainable material design and agricultural preservation. As illustrated in Figure 15, irradiation induces the formation of free radicals that initiate crosslinking or grafting reactions along SA molecular chains, enabling the development of biodegradable hydrogels and coating systems with enhanced structural integrity and functionality. Through this mechanism, SA-based materials can be precisely tailored for specific applications, offering improved mechanical properties, water absorption, and environmental stability. For example, a biodegradable SA-g-acrylamide/acrylic acid hydrogel has been developed via electron beam-induced graft copolymerization, where high-energy electrons initiate the formation of a three-dimensional (3D) crosslinked network on the SA chains, resulting in materials with superior water absorption and controllable degradation behavior [138]. Similarly, γ-irradiation-induced copolymerization enables tunable crosslinking density and improved swelling capacity of hydrogels in saline media [139]. The irradiation dose directly influences the balance between crosslinking and degradation, allowing fine modulation of gel strength and swelling ratio.

In agricultural preservation, SA coating combined with ^60^Co-γ irradiation has been shown to significantly extend the postharvest life of fruits such as jujube. The irradiation treatment enhances the semi-permeable nature of the SA film, reducing moisture loss and oxygen permeability while delaying nutrient degradation and microbial growth [140]. Furthermore, irradiation-assisted SA systems have been used to construct pH-responsive and superabsorbent hydrogels, offering sustainable solutions for soil moisture retention and crop protection. This residue-free and energy-efficient physical technique, when integrated with the inherent biocompatibility of SA, holds great promise for eco-friendly material development, sustainable agriculture, and biomedical engineering.

#### 4.2.3. Physical Crosslinking

SA synergistically constructs multifunctional hydrogel systems with tunable properties via dynamically reversible physical crosslinking mechanisms (e.g., ionic bonding, hydrogen bonding, and photochemical/chemical responsive interactions). For the hydrogel delivery system, SA was modified with glycerol methacrylate to introduce photosensitive groups and combined with photocrosslinking technology to create an NO-responsive hydrogel. In this system, carboxyl groups from SA and the NO-responsive agent form a dual crosslinked network through covalent and hydrogen bonds, resulting in low swelling rates and high temperature stability [141]. Regarding gels, SA was synergistically constructed with polyacrylamide (PAAm)/pozzolanic (Pal) nanorods [142] and gelatin (Gel) [143] to form high-strength composite hydrogels (SA-PAAm/Pal and Gel-Alg) via dynamic metal ion cross-linking. Additionally, dynamic hydrogels were also constructed with starch through the pH-responsive cross-linking mechanism induced by glucolactone (GDL) [144]. Further, oxidatively modified SA (OSA) formed physically crosslinked nanogels with neutral proteins (e.g., hemoglobin), assisted by divalent cations [145]. This design endowed the materials with self-recovery capability, enhanced matrix stiffness, and high flexibility and ionic conductivity, which synergistically reinforced the mechanical strength and thermal stability of gels. Alternatively, SA constructed mechanically enhanced biosponges (SA-BG) by ionic cross-linking with Ca^2+^ released from bioactive glass particles (RFNP-BG). This crosslinked network maximized the porous structure and provided enhanced water resistance, while the continuous release of Ca^2+^ imparted bioactivity to the material [146]. To end with, the carboxyl groups in SA are coordinated with metal ions, bound to reactive substances, and modulated in response to the network, collectively conferring self-healing, energy dissipation, and environmental adaptation properties.

### 4.3. Biological Modification

**Figure 16:**
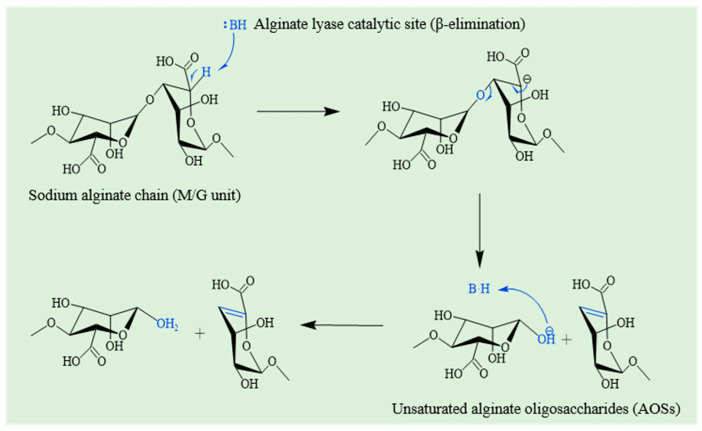
Schematic representation of the β-elimination catalytic mechanism of alginate lyase acting on SA, showing the enzymatic cleavage of 1,4-glycosidic linkages between uronic acid residues and the formation of unsaturated alginate oligosaccharides (AOSs).

The biomodification primarily refers to the controlled degradation and structural alteration of SA through biological methods to obtain target products with specific molecular weight, sequence structure, and desired bioactivity. Enzymatic modification [147] of alginate serves as the core strategy due to its high efficiency and substrate specificity. Compared to chemical methods, enzymatic modification provides several advantages [148], such as milder reaction conditions, higher specificity, and the production of well-defined bioactive oligosaccharides by cleaving 1,4-glycoside bonds through the β-elimination reaction. Sodium alginate lyase, as a key enzyme to realize this process, is produced by various organisms, including seaweeds, marine mollusks, fungi, viruses, as well as terrestrial and marine bacteria, with bacterial source being the most predominant [149]. These enzymes are categorized into different families based on their catalytic mechanisms and structures [150]. According to the Carbohydrate-Active enZYmes (CAZy) database, alginate lyase sequences are distributed across 12 polysaccharide lyase (PL) families (PL5, PL6, PL7, PL14, PL15, PL17, PL18, PL31, PL32, PL34, PL36, and PL39) [151]. The catalytic mechanism of alginate lyase acting on sodium alginate involves β-elimination of the 1,4-glycosidic linkage between uronic acid residues, leading to the formation of unsaturated alginate oligosaccharide (AOSs), as illustrated in Figure 16. This β-elimination process represents the general catalytic pathway of alginate lyases. According to the action mode, alginate lyases are classified as either endolytic or exolytic enzymes [152]. Endolytic lyases cleave internal bonds within the alginate polymer to generate oligosaccharides [153], while exolytic lyases further degrade oligosaccharides into monomers and/or dimers from the non-reducing ends [154]. Furthermore, alginate lyases are categorized into three groups based on their substrate specificities: poly(M)-specific lyases, poly(G)-specific lyases, and bifunctional lyases that can degrade both poly(M) and poly(G) [155]. These enzymes are widely employed to produce alginate oligosaccharides (AOSs) [156] with tailored bioactivities, demonstrating considerable potential for applications in the pharmaceutical and functional food industries [157].

### 4.4. Comparison of Modification Methods

Chemical modification involves the change in the chemical structure of SA through chemical reactions (such as esterification, oxidation, grafting, etc.). Physical modification, on the other hand, alters the properties of materials through physical actions (such as composite modification, physical cross-linking, etc.) without damaging the chemical structure. Biological modification is the process of regulating the structure of SA or complex bioactive compounds by biological approaches (e.g., enzymatic treatment) under relatively mild conditions. These three modification approaches—chemical, physical, and biological—are compared and analyzed in terms of modification effect, cost, process difficulty, toxicity risk, and application field, as summarized in Table 1.

## 5. Application of Modified SA

### 5.1. Food Industry

#### 5.1.1. Food Packaging

Food packaging materials [158] protect food from external contamination, inhibit deterioration [159], and extend shelf life [160]. High-quality packaging materials [161] can also facilitate storage, transport, and sale of food, safeguard food safety, enhance product value, and meet the needs of consumers for food hygiene, esthetics, and convenience [162]. Alginate excels in film-forming, creating films characterized by high tensile strength, flexibility, tear resistance, oil resistance, stiffness, and superb gloss [163]. For instance, tannic-acid-modified SA edible films show a concrete barrier/antioxidant gain: WVP drops from 1.24 × 10^−6^ to 0.54 × 10^−6^ g m/(h Pa), with ≈89% DPPH scavenging and ≈98% UV blocking at 280 nm [164]. Furthermore, alginate is odorless and has a neutral taste; its coatings possess antimicrobial properties that inhibit bacterial growth and oxidative odor formation [165]. In food applications, alginate coatings can significantly improve the organoleptic acceptability of products and reduce cooking losses [85]. As a representative case, an electrospun PVA/SA/PVDF bilayer indicator for pork gives a strong NH_3_ response (ΔE ≈ 48) and prolongs shelf life by ~24 h at 25 °C [166].

For biodegradable food packaging materials, SA can be compounded with cinnamic acid-modified pectin [167] or tannic acid (TA) [164]. Quantitatively, the SA–pectin–cinnamate film shows ≈43.26% soil mass loss at 15 d while maintaining plastic-like mechanics, and the TA–SA system retains mechanical integrity while lowering WVP as above. Both compounds form biodegradable films with indicators and performance comparable to polyethylene plastic films, indicating the potential to replace traditional plastics. In smart indication packaging [168], SA was composited with agar (AG) [169], PVA/alizarin [166], PVA/rosemary anthocyanins (RAs) [170,171], and casein carboxymethylcellulose nanocomplexes (CAS-CMC-ACNs) [172]. These films enabled real-time freshness monitoring [173], pH visualization [165], and dynamic spoilage tracking [174]. Zhang et al. [175] composited SA with PVA and metal–organic framework (ZIF-8) to create a colorimetric sensor (PA-SA-ZA) for high-precision visualization of beef freshness with ΔE < 5 under light-aging, contact angle ≈ 52°, and a color–TVB-N correlation R^2^ ≈ 0.91 [176].

**Figure 17:**
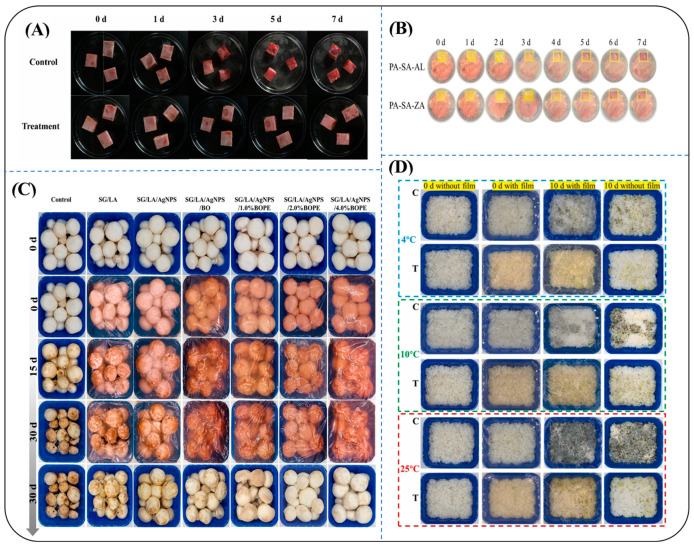
Representative applications of modified SA-based packaging for food preservation and freshness indication. (**A**) Active film incorporating carvacrol via a dialdehyde β-cyclodextrin/gelatin–carrageenan network for ready-to-eat meat preservation (quality retention over 0–7 days) [160]; (**B**) Ammonia-responsive colorimetric indicator (PA-SA-ZA; PVA/SA with ZIF-8-alizarin) tracking beef freshness over storage days [175]; (**C**) SA/guar-gum (SG) film combined with BOPE Pickering emulsion preserving mushrooms during cold storage (0–30 days) [184]; (**D**) GG/AG/SA bilayer incorporating Pickering emulsion and TiO_2_, exhibiting antifungal and barrier enhancement at 4, 10, and 25 °C (comparison with and without film) [186].

For active packaging, SA can be laminated with cyclobutanedicarboxylic acid (CBDA-10) [177], gelatin (GEL)/waste green tea extract (GTE) [178], and curdlan (CD) [163]. For example, CBDA-crosslinked SA films report TS ≈ 148 MPa, Td ≈ 249 °C, and ~60% soil mass loss at 4 weeks; curdlan–SA films improve mushroom (Volvariella volvacea) shelf life with reduced microbial load and firmer texture. These bioactive films can strengthen the mechanical strength and stability of the film while providing highly efficient dual-functionality (antimicrobial freshness preservation). To better illustrate the diversity of food-compatible SA-based packaging systems, Table 2 summarizes representative formulations, modification routes, and their corresponding performance indices (e.g., barrier, antioxidant, and antimicrobial properties). Figure 17 further visualizes their practical applications in meat, mushroom, and produce preservation, highlighting both active and intelligent functionalities. Concerning nanomaterials [179], SA can be blended with polyethylene oxide (PEO)/leaf skin tannin (Ph) [180] or complexed with pullulan polysaccharide [181] to produce nanofiber films for targeted antimicrobial protection and enhanced mechanical strength [182] and moisture barriers [183] of the films. Furthermore, sodium alginate (NaAlg) was used as a nanoencapsulation matrix for bovine lactoferrin (LFb) into nanoparticles (LFNP) and microcapsules via high-voltage electrohydrodynamic atomization (EHDA) technology. The EHDA process yields ~100–200 nm particles with |ζ| ~ 20 mV, enabling controlled stabilization/release for iron delivery in functional packaging.

Regarding other materials, SA was blended with guar gum to form a composite film (SG), where mechanical strength and water resistance were synergistically enhanced by incorporating a β-cyclodextrin/persimmon pectin-stabilized baobab oil-peeled Kleenex emulsion (BOPE) [184]. Based on this, SA was synergistically combined with guar gum and agar to construct a bilayer composite film. Within this structure, the carboxylic acid groups of SA enhanced the densification of polysaccharide network through hydrogen bonding, improving the mechanical strength and moisture resistance of the film [185], thereby achieving dual optimization of “structure-function” for food packaging [186]. Notably, several chemical modification routes relevant to food-contact applications rely on food-compatible reagents or bio-based crosslinkers (e.g., phenolic acids such as tannic/cinnamic acids, genipin or enzyme-mediated coupling), which improve barrier/mechanical/antimicrobial performance while maintaining compliance pathways typical for edible films and food-contact materials. These uses are distinguished from non-food biomedical/environmental chemistries summarized elsewhere in this review. In the ultrasound-assisted preparation of soybean protein isolate-based packaging materials, SA effectively inhibits the formation of lysinoalanine (LAL) by promoting protein conformational crosslinking [187].

In summary, SA significantly improves the antibacterial, antioxidant, and UV-barrier properties of biodegradable food packaging materials, thereby extending the shelf life of fresh foods and enabling visualization and monitoring of spoilage. These integrated environmentally friendly–functional–intelligent characteristics provide a technological foundation for the green and intelligent transformation of the food packaging industry.

**Table 2:** Representative modified SA-based films for food packaging.

System	Modification	Food Matrix	Key KPl/Value	Condition/Note	Reference
Curdlan–SA active film	Polysaccharide blend	Volvariella volvacea (mushroom)	Shelf-life ↑; microbial load ↓; firmness retained	Cold storage	[163]
SA–Pectin + Cinnamic Acid	Phenolic ester (active, biodegradable)	General	≈43.26% soil mass loss at 15 d; plastic-like mechanics	Soil burial vs. PE	[167]
SA + Tannic Acid (TA) edible film	Phenolic crosslinking	Produce/Meat (general)	WVP 1.24 × 10^−6^ → 0.54 × 10^−6^ g·m/(h·Pa); DPPH ≈ 89%; UV-block ~ 98%@280 nm	Lab films; edible/food-contact	[164]
PVA/SA/PVDF bilayer (alizarin sensor + antibacterial top)	Electrospun bilayer indicator	Pork	ΔE ≈ 48 (NH_3_); shelf-life +~24 h @25 °C	Pack test	[166]
PVA–SA + ZIF-8@alizarin (PA-SA-ZA)	MOF-stabilized dye sensor	Beef	ΔE < 5 under light aging; R^2^ ≈ 0.91 (TVB-N vs. color); contact angle ~ 52°	Pack test	[175]
PEO/SA nanofiber + phlorotannin	Electrospun antimicrobial	Chicken	Salmonella counts ↓; shelf-life ↑	Cold storage	[180]
SA/Guar Gum + BOPE Pickering film	β-CD/persimmon pectin-stabilized oil emulsion	Mushrooms	Browning/shrinkage ↓; water/oxygen ingress ↓	Postharvest	[184]
GG/AG/SA bilayer + TiO_2_	Bilayer + Pickering + nanofillers	High-moisture produce	Barrier ↑; antifungal ↑	Postharvest	[186]

Abbreviations: SA, sodium alginate; TA, tannic acid; PVA, poly(vinyl alcohol); PVDF, poly(vinylidene fluoride); ZIF-8, zeolitic imidazolate framework-8; GG, guar gum; AG, agar; BOPE, β-cyclodextrin/persimmon-pectin-stabilized baobab-oil Pickering emulsion; WVP, water vapor permeability; TVB-N, total volatile basic nitrogen; SGF/SIF, simulated gastric/intestinal fluid; EE%, encapsulation efficiency; arrows (↑/↓) indicate direction of change.

#### 5.1.2. Functional Food Carrier

Functional food ingredients [188] require suitable delivery systems (or carriers) [189] to accommodate their diverse physicochemical and biological characteristics. Such carriers are essential for protecting bioactive molecules during processing and gastrointestinal transit, ensuring that they reach their intended target sites. SA is a core carrier material for functional active ingredient delivery systems, owing to its biocompatibility, controllable gelation, and designability of multi-scale structural design [190]. For lipophilic antioxidants (e.g., α-tocopherol), SA microcapsules provide gastric protection with intestinal release: cumulative release is ~29% in SGF but ~82% in SIF, with T_50_% ≈ 3.8 h and T_70_% ≈ 12.3 h (SIF) [191].

For microencapsulation, SA has been combined with whey protein isolate (WPI) [192], pectin [191], chitosan [193], and gelatin [194], as wall materials. In SA–WPI ionic-gel systems, formulation knobs (polymer ratio and Ca^2+^ dosage) tune encapsulation efficiency (EE%) and bead size; typical working windows used for food-grade beads are SA ≈ 0.8–1.5% (*w*/*w*), WPI ≈ 1–3%, and CaCl_2_ ≈ 20–60 mM, which can be optimized through response surface design to balance an increase in EE%, a reduction in particle size, and a decrease in burst release [192]. These SA-based composites demonstrate efficient encapsulation and structural control of active ingredients, optimizing both microcapsule architecture and encapsulation performance while providing high stability.

Moreover, SA plays a critical protective role in probiotic targeting delivery by forming a dual network gel (FSDN) with fish gelatin (FG) [195]. Through ionic gelation (internal or external) [196], SA forms stable networks capable of withstanding harsh gastrointestinal conditions (e.g., gastric acid, bile salts), thereby ensuring probiotic viability and controlled intestinal release. Quantitatively, FG/SA-DN capsules increase encapsulation efficiency from ~16% to ~92% as FG increases [195] and can raise acid-stage survival to ~83.6% in SGF compared with significantly lower survival of free cells [196]. SA-based microcapsules formed by combining proteins, polysaccharides, or dual-network systems exhibit notable advantages in encapsulation efficiency, targeted release, and environmental tolerance, effectively preserving essential oils, fat-soluble nutrients, probiotics, and other sensitive bioactives. To summarize representative SA carrier systems and their key physicochemical–functional readouts, Table 3 outlines typical encapsulation matrices, mechanisms, and food-oriented applications.

Beyond ionic gelation, mild chemical tailoring using food-compatible reagents—such as phenolic crosslinking or peptide/amino acid coupling—can further stabilize alginate carriers while maintaining their suitability for ingestion or food-contact use under standard migration and safety assessments. Practically, phenolic (e.g., tannic/cinnamic) crosslinking can be performed at ≤45 °C in aqueous media with pH 5–7, followed by Ca^2^⁺ setting to co-lock the network; enzyme-assisted (laccase/tyrosinase) routes allow low-temperature curing for heat-sensitive cargos while limiting residuals to food-compatible species. These food-oriented chemistries are explicitly distinguished from stronger non-food reactions described in biomedical and environmental contexts.

### 5.2. Biomedical Application

#### 5.2.1. D Printing

SA is a low-cost and biocompatible biomaterial characterized by rapid and mild cross-linking, which has led to its widespread use in biological soft tissue repair and regeneration. Especially with the advent of 3D bioprinting technology, SA hydrogels have become increasingly popular in tissue engineering as a result of their exceptional printability [198]. Compared with traditional 2D approaches, 3D printing overcomes the restrictions of planar structure through a layer-by-layer stacking strategy, offering significant advantages in constructing bone tissue engineering scaffolds. Based on CT/MRI image data from patients with bone defects, 3D printing enables precise bionic design of personalized scaffolds, ensuring that both the macroscopic morphology and microscopic pore structure closely match natural bone tissues. Accordingly, it addresses the problems inherent to 2D printing, such as uneven distribution of cells and poor mechanical suitability resulting from the inability to construct complex 3D interconnected pore channels. The multilevel structural controllability of 3D printing not only promotes the directional migration of osteoclasts and vascularization, but also facilitates the mechanical gradient in scaffolds. By mimicking the stress-transferring properties of bone trabeculae through these designed gradients, 3D printing significantly improves the efficiency of bone integration [199]. In layered mesoporous bioactive glass/sodium alginate–sodium alginate (MBG/SA–SA) scaffolds, the printed architecture delivers porosity of ≈78% and a compressive strength of ≈4.2 MPa, matching cancellous-bone-level requirements and enabling fast release of BSA from the SA layer while maintaining sustained release of ibuprofen from the MBG/SA layer [200].

**Figure 18:**
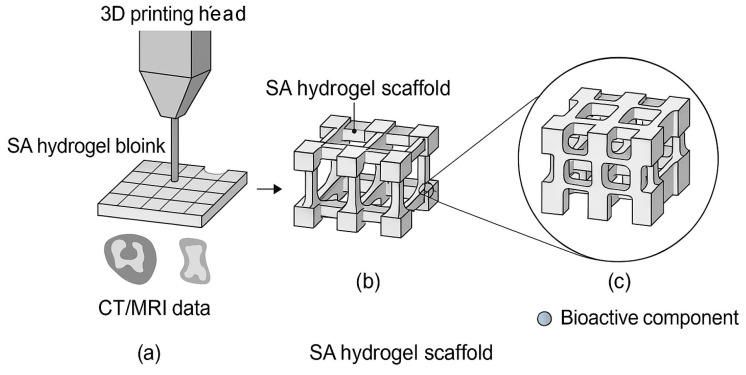
Schematic illustration of the 3D printing process using SA-based bioinks for tissue engineering scaffolds. (**a**) Layer-by-layer 3D printing of SA hydrogel guided by patient CT/MRI data; (**b**) formation of SA hydrogel scaffolds with interconnected porous microstructures; (**c**) incorporation of bioactive components such as mesoporous bioactive glass (MBG) or nacreous powder (NP) within the printed scaffold to enhance biological functionality.

Liu et al. [201] utilized SA as a core component of 3D printed bioink, composited with nacreous layer powder (NP) to construct bionic bone scaffolds, demonstrating the benefit of precise structure-function synergy in bone tissue engineering. Similarly, Song et al. [202] constructed a drug-carrying bilayer scaffold (SG-rhEGF) by compositing SA with gelatin, achieving precise structure-function regulation in the field of skin regeneration. Quantitatively, this bilayer shows elongation at break 102.09 ± 6.74% and tensile modulus 206.83 ± 32.10 kPa, with a hydrophobic outer layer (water contact angle 112.09 ± 4.67°) and hydrophilic inner hydrogel layer (48.87 ± 5.52°), supporting barrier function and moist-wound healing. Additionally, in bone repair, SA was incorporate with MBG to form a layered double network scaffold (MBG/SA-SA), exhibiting an integrated structural-functional design (see the above porosity/strength and dual-release metrics for process targeting and benchmarking in future studies) [200]. An illustrative schematic of the 3D printing process and scaffold design is shown in Figure 18, highlighting the layer-by-layer stacking strategy, hierarchy structure, and integration of SA-based materials with functional bioactive components.

#### 5.2.2. Drug Delivery Systems

SA is one of the most widely used natural polymers in drug delivery and biomedical applications. It functions as a gelling, thickening, and tablet disintegrating agent and has gained prominence as a cross-linking agent and for pH-controlled delivery. Due to its pH/ionic responsiveness and programmable crosslinking mechanism, SA is an important vehicle for multifunctional drug delivery systems. Alginate acts as a rate-controlling polymer in drug delivery, forming gels when hydrated with water under mild conditions without organic solvents. The resulting hydrogels formed were relatively inert, containing only distilled water or sucrose solutions [203]. Veronica et al. [204] employed SA as a natural pH-responsive carrier in oral slow-release formulations through ionic cross-linking and gelation mechanisms for controlled drug release. SA, as a polyanionic polysaccharide, can be complexed with cellulose nanocrystals (CNW). As a polyanionic polysaccharide, SA can form polyelectrolyte complexes (PECs). It is complexed with cellulose nanocrystals (CNCs) [205] to create PEC hydrogels for vaginal drug delivery, demonstrating precise environmental suitability. Similarly, SA complexed with carboxymethyl chitosan (CMC) [206] to form PEC sponges, which exhibited efficient synergistic effects in hemostatic materials. These applications highlight the smart responsiveness and multifunctional biomedical potential of SA-based PECs.

Reddy et al. [207] used SA as an ion-responsive carrier to develop a double crosslinked microbead with montmorillonite (MMT) for efficient loading and controlled release of hydrophobic drugs (e.g., curcumin CUR). The beads exhibited strong pH-gated behavior: at pH 7.4 the swelling degree rose to ~670% (CaMg formulation) within ~30–40 min versus ~110% at pH 1.2 (120 min), and CUR release reached ~68% at pH 7.4 vs. ~30% at pH 1.2 by ~1000 min; release followed a Korsmeyer–Peppas mechanism. Lin et al. [208] employed SA as a nanogel matrix to form a core–shell composite carrier by embedding liposomes, achieving a synergistic stability enhancement and controlled-release functionality. Cross-linking an internal alginate network increased liposomal particle rigidity by ~3 times, doubled blood circulation time, and enhanced drug accumulation in arthritic joints without altering surface properties. SA formed a 3D network in the core of liposomes by cross-linking with Ca^2+^ ions, providing mechanical support and reducing drug leakage rate, and preserving the surface properties of the liposomes to achieve target recognition.

**Figure 19:**
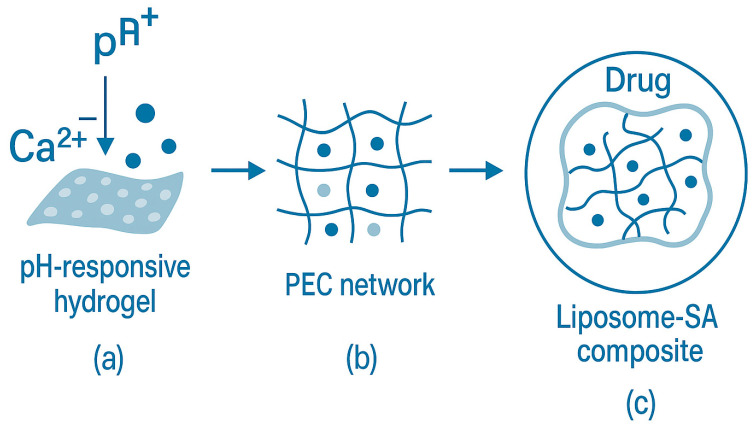
Schematic illustration of SA-based multifunctional drug delivery systems. (**a**) Formation of pH/ion-responsive hydrogels through Ca^2+^ crosslinking under acidic or basic conditions, enabling controlled drug release; (**b**) Construction of polyelectrolyte complex (PEC) networks between SA and oppositely charged polymers for enhanced drug encapsulation and stability; (**c**) Development of liposome–SA composite systems, where liposomes are coated with SA hydrogels to improve mechanical strength and achieve sustained release performance.

In summary, SA-based carriers efficiently regulate drug release kinetics via dynamic ionic bonding, polyelectrolyte complexation, or dual-network construction. This enables gastric acid barrier breakthrough, intestinal-targeted slow release, and focal microenvironmental response. An illustrative schematic of SA-based multifunctional drug delivery systems is presented in Figure 19, highlighting the pH/ion-responsive hydrogel formation, the construction of PEC networks, and the liposome–SA composite carriers for controlled drug release and enhanced stability.

### 5.3. Environmental Engineering

Sodium alginate and its derivatives exhibit significant potential in environmental remediation. Their surfaces are rich in hydroxyl (-OH) and carboxyl (-COOH) functional groups that can efficiently capture dye molecules, heavy metal ions, and organic pollutants [209] through ion exchange, electrostatic interactions, and coordination mechanisms. To improve adsorption performance, SA is commonly compounded with functional materials (e.g., activated carbon, graphene oxide, biochar, carbon nanotubes, etc.), and either physically embedded or chemically crosslinked. This creates a porous structure while introducing specific adsorption sites. Furthermore, cross-linking modification augments the spatial distribution of functional groups, significantly improving the stability and recyclability of materials. The environmentally friendly properties of these materials are derived from the biodegradability and renewability of their natural sources, offering innovative solutions for green water treatment technologies.

#### 5.3.1. Metal Ion Adsorption

**Figure 20:**
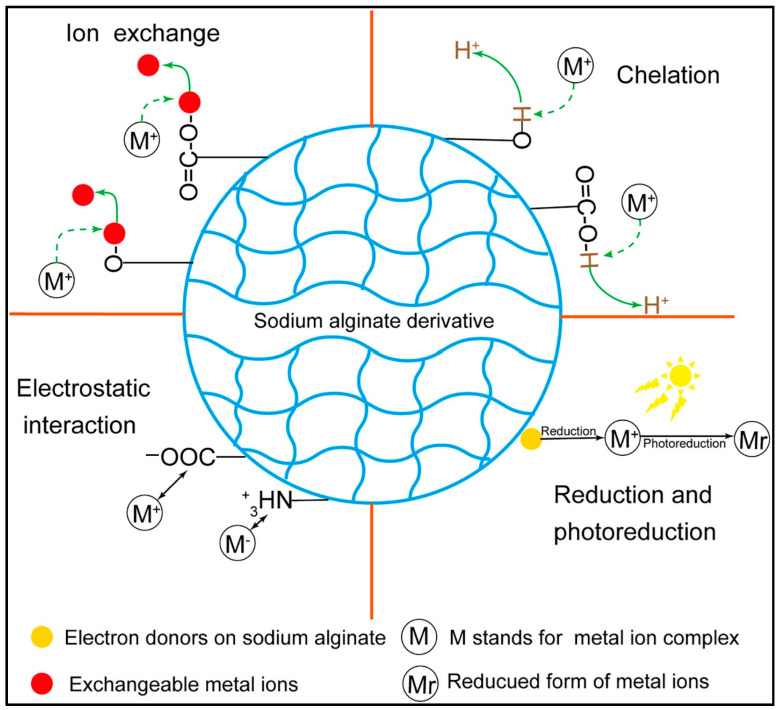
Adsorption mechanism of metal ions by SA-based adsorbent [215].

With rapid industrialization, heavy metal pollution [210] has become a major global environmental challenge [211]. Common heavy metal ion removal processes [212] include membrane separation, electrochemical recovery, chemical precipitation, and ion-exchange, as summarized in Table 4. In addition to these conventional techniques [213], functionalized SA-based adsorbents have recently attracted significant attention owing to their tunable surface chemistry, biocompatibility, and environmental sustainability. The key advantages and limitations of both traditional methods and SA-based functionalized systems are compared in Table 4. As a naturally occurring anionic polysaccharide, the carboxyl (-COOH) and hydroxyl (-OH) functional groups in the SA molecular chain can specifically bind to heavy metal ions via coordination, ion exchange, and electrostatic adsorption [214]. SA-based adsorbents exhibit high adsorption capacities for heavy metal ions, and their adsorption mechanism [215] is depicted in Figure 20. Composites of SA and PVA form Fe^0^-Fe_3_O_4_ nanocomposite beads [216], which demonstrate enhanced physical properties and catalytic reactivity in Cr(VI) removal. Under an optimized bead formulation (5.0 wt% PVA, 1.5 wt% SA with acidification/reduction), only 0.075 wt% Fe^0^ with 0.30 wt% Fe_3_O_4_ was sufficient to completely remove 20 mg·L^−1^ Cr(VI); the removal efficiency decreased from 100% to 79.5% as the initial Cr(VI) rose from 5 to 40 mg·L^−1^, and from 99.3% to 76.3% as pH increased 3.0 to 11.0; after four reuse cycles the beads retained 69.8% efficiency. Therefore, researchers incorporated graphene oxide (GO) into this system construct to develop 3D hydrogel microspheres (SPGs) [217], exhibiting efficient synergistic adsorption for heavy metal ions. Encapsulating magnetite-GO composites with SA produced core–shell structure adsorption microbeads (mGO/beads) [218], providing dual advantages of efficient adsorption and convenient recovery. Furthermore, SA is loaded onto melamine sponge (MS) via an in situ gelation process, creating composite adsorbents (alginate-MS) [219], significantly enhancing mechanical strength and cyclic stability while achieving a balance between adsorption efficiency and structural robustness.

Overall, the incorporation of sodium alginate not only enhances the adsorption efficiency of metal ions in wastewater but also provides a scalable and renewable solution for green and efficient heavy metal removal.

#### 5.3.2. Dye Wastewater Treatment

Synthetic dyes are among the most persistent water pollutants, posing dual threats to aquatic ecosystems due to their distinct physicochemical properties. Even at low concentrations, they can cause severe sensory pollution and disrupt aquatic food chains via bioaccumulation. These color-revealing compounds (cationic, anionic, and non-ionic) are extensively used in industries such as textiles, paper, plastics, and leather manufacturing. According to the United Nations Environment Programme (UNEP), globally, approximately 280,000 tons of dyestuffs enter the aquatic environments annually through industrial wastewater. A small portion of these dyestuffs resists biodegradation due to their stable molecular structure, forming persistent pollutants. Importantly, industrial dyes like triphenylmethane and azo types reduce water light transmittance, and their metabolic intermediates (e.g., carcinogenic aromatic amines) exhibit teratogenicity, highlighting the urgency of pollution control [230]. SA can be incorporated with TEMPO oxidized cellulose (TOC) [231] and PVA/starch [232] to construct composite gels. SA enables a novel photocatalytic-biological synergistic system (ICPB) by coupling R. palustris with carbon nanotubes-silver-modified titanium dioxide (CNT-Ag-TiO_2_) photocatalysts [233], overcoming the limitations of the stability of traditional adsorbents. Additionally, a composite photocatalytic material (Cu-BTC @Alg/Fe_3_O_4_) was synthesized by integrating copper-based MOF (Cu-BTC) with magnetic Fe_3_O_4_ [234], addressing photocatalyst deactivation and secondary pollution, and providing an environmentally friendly treatment for highly toxic dyes. Quantitatively, Cu-BTC @Alg/Fe_3_O_4_ exhibited a BET surface area of ~160 m^2^·g^−1^, an adsorption capacity of ~200 mg·g^−1^ for Rhodamine B with ~97% removal, achieving equilibrium within ~100 min and following pseudo-second-order kinetics (R^2^ ≈ 0.999) [233].

Overall, the incorporation of SA not only improves the adsorption efficiency of metal ions in wastewater but also provides a scalable and renewable solution for green and efficient heavy metal removal.

### 5.4. Other Applications

#### 5.4.1. Smart Textiles

The rapid development of flexible electronics is propelling smart fiber research into a new phase, focusing on the construction of wearable electronic platforms with inherent flexibility and system integration through textile-based components. The global smart textiles market is predicted to increase remarkably, thanks to breakthroughs in woven smart fibers in thermal management, electromagnetic protection, and bioelectrical signal coupling. Such fiber-level systems achieve conformal contact with the curved surfaces of the human body, providing superior breathability and biocompatibility than traditional rigid electronics [235]. SA builds multifunctional fibers by compositing with liquid metal (LM) micro/nanodroplets through a wet-spinning process [236], and also modifies cotton nonwoven fabrics in synergy with silver nitrate via the sol–gel method [237]. Practically, an SA–Ag system using 1 wt% sodium alginate and 15 wt% AgNO_3_ achieves surface resistivity < 100 Ω sq^−1^ on cotton nonwovens, meeting conductive-textile requirements while retaining textile handle [237]. These innovations deliver breakthrough material properties in smart textiles. The composite system maintains the high air permeability and flexibility of the material, while the ionic cross-linking network of SA imparts self-repairing properties, providing a solution for the human–machine interfaces in extreme environments.

#### 5.4.2. Microbial Fuel Cell

Alginates and their derivatives have emerged as promising alternative materials for microbial fuel cells. Due to their unique physicochemical properties, these natural polymers are particularly suitable for various fuel cell systems, including polymer biofuel cells, electrolyte fuel cells, polymer electrolyte fuel cells, and direct methanol fuel cells. In these applications, alginates and their compounds can effectively replace conventional proton exchange membranes, demonstrating strong performance and application prospects [85]. SA can be composited with polyaniline (PANI) [238], agar-activated carbon (AC) [239], or super-activated carbon (SAC) [240] to fabricate self-supporting anodes, achieving synergistic enhancement in electron storage and energy conversion in microbial fuel cells (MFCs). This induced dual optimization of electrode performance and biocompatibility, notably optimizing the microbial immobilization and electron transfer efficiency. Additionally, SA maintains stable power generation in complex wastewater environments through a two-layer hydrogel structure [241], i.e., the inner layer of SA and Fe_3_O_4_ to form a 3D porous network, and the outer layer of SA covalently cross-linking with PVA to form a dense protective layer. Under high-salinity waste-leachate feeding, this double-layer SA bioanode delivered an open-circuit voltage (OCV) ~ 1.17 V and an operating voltage ~ 781 mV, evidencing salt-resistant, long-term stable output. This “functional gradient” bioelectrode development is for high salt/high bacterial load wastewater. Additionally, SA can be compounded with PVA/chitosan to form a functionalized ion-conducting layers that optimize electrode interfaces and electrical output performance in MFCs [239].

#### 5.4.3. Soil Conditioner

Improper application of chemical fertilizers (e.g., unsuitable timing or dosage) leads to nutrient loss, soil degradation [242], and water pollution [243], contradicting modern environmental protection principles. Although various soil amendments are widely employed in agricultural, natural polymers have recently gained popularity for improving the utilization of fertilizers [244]. Among these, SA has become a key functional material in soil remediation and sustainable agriculture due to its biodegradability, dynamic cross-linking ability, and multiscale structural designability [245]. SA can be compounded with sulfide-modified iron nanoparticles [246], biochar [247], and gelatin [248] to construct a soil conditioner or hydrogel, achieving the synergistic effect of “degradation-passivation-structural” improvement in soil pollution control, and the multifunctional integration of “carrier-nutrient-improvement” for intelligent slow-release fertilizers. Specifically, for SA/sulfide-coated Fe nanoparticles activating persulfate in contaminated soil, response-surface optimization indicated a theoretical 99.79% TBBPA degradation at 34.28 °C using 3.57 g·kg^−1^ SA@S-Fe and 36.35 mM persulfate, while heavy metals (Fe, Cu, Zn) remained in stable residual fractions and soil SOM/TN/C/N/TOC only slightly decreased [246]. Furthermore, SA can capture organic pesticides and inhibit their migration and diffusion via electron beam irradiation grafting [138] and amphiphilic modification technology [249]. This addresses the residual pollution from traditional improvers, while providing environmentally friendly solutions for water-saving agriculture and precision fertilization. In summary, SA-based system, through nanoparticle compositing, biochar integration, or amphiphilic modification, achieve multi-effect synergy by targeted degradation of pollutants, intelligent slow release of nutrients, and optimization of soil microstructure. This approach overcomes the limitation of traditional single-function and residual pollution of soil conditioners, providing effective solutions for green agriculture and ecological restoration.

## 6. Conclusions and Outlook

This review highlights that the modification of sodium alginate should be viewed within a food-oriented and food-contact framework, rather than as the development of new food additives. Various modification strategies—including chemical tailoring, physical processing, and enzymatic regulation—have effectively expanded the functional potential of SA while maintaining its safety and biocompatibility for edible and food-related applications.

Food-compatible chemical routes (e.g., phenolic, enzymatic, and genipin crosslinking) and green physical techniques (e.g., ultrasound treatment, irradiation, and biopolymer blending) enable the fabrication of biodegradable films, coatings, and delivery systems that meet both functional and regulatory requirements. These mild modification approaches enhance mechanical strength, barrier performance, and antimicrobial activity, supporting the development of active and intelligent food packaging and functional ingredient carriers.

Beyond food applications, insights derived from non-food-oriented modification studies (such as graft copolymerization or nanoparticle incorporation) can guide the design of next-generation bio-based materials with tailored structure–function relationships. Future research should focus on integrating molecular modification mechanisms with performance evaluation in real food systems, while advancing scalable, low-residue, and life cycle-optimized production processes. Through interdisciplinary efforts linking food science, materials chemistry, and sustainable engineering, sodium alginate is expected to serve as a core platform for safe, multifunctional, and eco-friendly food systems.

## Figures and Tables

**Table 1:** Comparison of chemical, physical, and biological modification methods for SA.

Dimension	Chemical Modification	Physical Modification	Biological Modification
Modification effect	Strong functionalization and high stability	Simple, fast, and environmentally friendly	High biocompatibility and strong specificity
Cost	High (reagents, purification)	Low (no need for complex equipment)	Extremely high (enzyme/genetic engineering)
Technological difficulty	Complex (requiring precise control of reaction conditions)	Simple (easy to industrialize)	Complex (requiring biotechnological conditions)
Toxic risk	Harmful substances may remain	None	None
Application	Industrial adsorbents and functional materials	Food packaging, sustained-release carriers	Biomedical, tissue engineering

**Table 3:** SA-based functional carriers: methods and readouts.

Cargo/System	Matrix and Method	Key Readouts	Use-Case	Reference
Bovine lactoferrin (LFNP)	EHDA nanoparticles in NaAlg matrix	Size ~ 100–200 nm; |ζ| ~ 20 mV; stable dispersions	Iron-delivery/antioxidant	[197]
Marjoram essential oil (EO)	SA + WPI (ionic gel)	EE/size tuned by SA/WPI/Ca^2+^; stable aroma retention	Antimicrobial flavor delivery	[192]
α-Tocopherol	SA beads (ionic gel)	Release ~ 29% (SGF) vs. ~82% (SIF); T50% ~ 3.8 h; T70% ~ 12.3 h (SIF)	Gastric protection; intestinal delivery	[191]
Probiotics (*Lactobacillus* spp.)	Fish-gelatin/SA double-network (FG/SA-DN)	Encapsulation efficiency ~16%→~92% (FG ↑); GI/thermal survival ↑	Fermented/baked foods	[195]

Abbreviations: arrows (↑/↓) indicate the direction of increase or decrease in the measured parameter.

**Table 4:** Comparative summary of conventional heavy metal ion removal techniques and functionalized SA-based adsorption systems.

Technology	Key Advantages	Major Limitations	Reference
Membrane separation	Minimal chemical usage, compact system footprint, selective metal recovery	High membrane procurement cost, frequent fouling issues, restricted throughput capacity	[220]
Electrochemical recovery	High-purity metal recovery, ambient condition compatibility	Intensive energy demand, slow reaction kinetics, potential electrolyte contamination	[221]
Chemical precipitation	Simplified operational workflow, low infrastructure cost	Excessive sludge yield (high disposal burden), non-selective removal, risk of secondary contamination	[222]
Ion-exchange	Targeted metal binding capability, high regeneration efficiency	Elevated upfront investment, narrow pH operating range, recurrent maintenance expenses	[223]
SA (Ca^2+^-crosslinked) hydrogel beads	Abundant carboxylate groups for chelation; low cost; biocompatible; easy beadization; regenerable with mild eluents	Gel swelling/softening; dissolution at low pH or chelating eluents; limited selectivity; intraparticle diffusion limits	[224]
Magnetic SA/Fe_3_O_4_ beads	Rapid magnetic separation; easy recovery and reuse; good dispersion	Fe_3_O_4_ oxidation/leaching; capacity decay across cycles; acid instability; added material cost	[225]
SA–biochar/zeolite/clay hybrids	Low-cost supports; improved permeability and strength; resilience to turbidity	Batch-to-batch variability; competing ions; fines shedding	[226]
Thiol-functionalized SA (–SH, dithiocarbamate)	High selectivity for soft metal ions (e.g., Hg^2+^, Ag^+^, Pb^2+^, Cd^2+^)	Thiol oxidation; odor; multi-step synthesis; cost	[227]
Amine/EDA/PEI-functionalized SA	Strong complexation with Cu^2+^/Ni^2+^/Cr (VI); rapid kinetics	Amine protonation at low pH reduces capacity; polymer leaching; fouling	[228]
Phosphate/phosphonate-modified SA	High affinity for Pb^2+^, rare earths; improved selectivity in competing electrolytes	Synthesis complexity; potential ligand leaching; cost	[229]

## Data Availability

No new data were created or analyzed in this study. Data sharing is not applicable to this article.
